# Risk Factors Associated With Nonfatal Opioid Overdose Leading to Intensive Care Unit Admission: A Cross-sectional Study

**DOI:** 10.2196/32851

**Published:** 2021-11-08

**Authors:** Avijit Mitra, Hiba Ahsan, Wenjun Li, Weisong Liu, Robert D Kerns, Jack Tsai, William Becker, David A Smelson, Hong Yu

**Affiliations:** 1 College of Information and Computer Sciences University of Massachusetts Amherst Amherst, MA United States; 2 Department of Public Health University of Massachusetts Lowell Lowell, MA United States; 3 Center for Healthcare Organization and Implementation Research Veterans Affairs Bedford Healthcare System Bedford, MA United States; 4 Department of Computer Science University of Massachusetts Lowell Lowell, MA United States; 5 Department of Psychiatry Yale University School of Medicine New Haven, CT United States; 6 Department of Neurology Yale University School of Medicine New Haven, CT United States; 7 Department of Psychology Yale University School of Medicine New Haven, CT United States; 8 Pain Research, Informatics, Multimorbidities and Education Center Veterans Affairs Connecticut Healthcare System West Haven, CT United States; 9 School of Public Health University of Texas Health Science Center at Houston Houston, TX United States; 10 National Center on Homelessness Among Veterans United States Department of Veterans Affairs Tampa, FL United States; 11 Department of Internal Medicine Yale University School of Medicine New Haven, CT United States; 12 Department of Psychiatry University of Massachusetts Chan Medical School Worcester, MA United States; 13 Department of Medicine University of Massachusetts Chan Medical School Worcester, MA United States

**Keywords:** opioids, overdose, risk factors, electronic health records, social and behavioral determinants of health, natural language processing, intensive care unit

## Abstract

**Background:**

Opioid overdose (OD) and related deaths have significantly increased in the United States over the last 2 decades. Existing studies have mostly focused on demographic and clinical risk factors in noncritical care settings. Social and behavioral determinants of health (SBDH) are infrequently coded in the electronic health record (EHR) and usually buried in unstructured EHR notes, reflecting possible gaps in clinical care and observational research. Therefore, SBDH often receive less attention despite being important risk factors for OD. Natural language processing (NLP) can alleviate this problem.

**Objective:**

The objectives of this study were two-fold: First, we examined the usefulness of NLP for SBDH extraction from unstructured EHR text, and second, for intensive care unit (ICU) admissions, we investigated risk factors including SBDH for nonfatal OD.

**Methods:**

We performed a cross-sectional analysis of admission data from the EHR of patients in the ICU of Beth Israel Deaconess Medical Center between 2001 and 2012. We used patient admission data and International Classification of Diseases, Ninth Revision (ICD-9) diagnoses to extract demographics, nonfatal OD, SBDH, and other clinical variables. In addition to obtaining SBDH information from the ICD codes, an NLP model was developed to extract 6 SBDH variables from EHR notes, namely, housing insecurity, unemployment, social isolation, alcohol use, smoking, and illicit drug use. We adopted a sequential forward selection process to select relevant clinical variables. Multivariable logistic regression analysis was used to evaluate the associations with nonfatal OD, and relative risks were quantified as covariate-adjusted odds ratios (aOR).

**Results:**

The strongest association with nonfatal OD was found to be drug use disorder (aOR 8.17, 95% CI 5.44-12.27), followed by bipolar disorder (aOR 2.69, 95% CI 1.68-4.29). Among others, major depressive disorder (aOR 2.57, 95% CI 1.12-5.88), being on a Medicaid health insurance program (aOR 2.26, 95% CI 1.43-3.58), history of illicit drug use (aOR 2.09, 95% CI 1.15-3.79), and current use of illicit drugs (aOR 2.06, 95% CI 1.20-3.55) were strongly associated with increased risk of nonfatal OD. Conversely, Blacks (aOR 0.51, 95% CI 0.28-0.94), older age groups (40-64 years: aOR 0.65, 95% CI 0.44-0.96; >64 years: aOR 0.16, 95% CI 0.08-0.34) and those with tobacco use disorder (aOR 0.53, 95% CI 0.32-0.89) or alcohol use disorder (aOR 0.64, 95% CI 0.42-1.00) had decreased risk of nonfatal OD. Moreover, 99.82% of all SBDH information was identified by the NLP model, in contrast to only 0.18% identified by the ICD codes.

**Conclusions:**

This is the first study to analyze the risk factors for nonfatal OD in an ICU setting using NLP-extracted SBDH from EHR notes. We found several risk factors associated with nonfatal OD including SBDH. SBDH are richly described in EHR notes, supporting the importance of integrating NLP-derived SBDH into OD risk assessment. More studies in ICU settings can help health care systems better understand and respond to the opioid epidemic.

## Introduction

The opioid epidemic in the United States is one of the most severe public health emergencies in recent times, with opioid overdose (OD) deaths quadrupling from 1999 to 2019 [1]. Almost 50,000 OD-related deaths occurred in 2019 alone [2], and the estimated economic burden including opioid use disorder and fatal OD totaled US $1021 billion during 2017 [3]. The sharp rise in opioid fatalit is responsible for a decline in the US life expectancy [4] and a surge in “deaths of despair” [5]. The opioid crisis is a complex situation involving a broad range of contributing factors including social determinants of health (SDOH) [6,7].

SDOH are the conditions in which people are born, live, work, and age [8]. Adverse SDOH can affect health through various means. For example, social or familial disruptions are well-known precipitants of suicide attempt [9-11]. Behavioral determinants include alcohol consumption, tobacco usage, and use of illicit drugs, among others. Together, adverse social and behavioral determinants of health (SBDH) can be defined as those variables that can hinder an individual’s disease management and negatively impact existing medical conditions [12]. Multiple prior studies suggested strong correlations between OD and a number of SBDH [6,7,13]. Analyzing SBDH in relation to OD can help us better address the OD crisis.

Prior studies found that lack of SBDH information can significantly decrease health care quality [14,15]. Realizing the impact of SBDH on health outcomes, many prior studies focused on extracting SBDH from structured data (eg, diagnosis codes, medications) and/or unstructured data (eg, discharge summaries, progress notes) [11,12,16-18]. However, existing electronic health records (EHRs) often lack the necessary SBDH information in a structured format, undermining its use in clinical care and research settings. On the other hand, EHR notes often describe SBDH [19], for example, financial insecurity (eg, “$807 SSI and $16/month food stamps*”*) and risky alcohol consumption (eg, “Drinking >4 drinks on one occasion or >14 drinks per week*”*). In addition, EHR notes describe change of status (eg, “recently lost job” or “recently purchased a gun”) that may more precisely identify the current state of a patient. As a consequence, we can take advantage of the rich information provided by unstructured EHR notes via natural language processing (NLP) [20]. NLP has already been successfully utilized for essential information extraction from EHR text to examine various clinical problems, including opioid use and risk assessment [21,22].

With nonfatal ODs increasing, there is a growing need for critical care of these patients in the United States [23]. Although a relatively high proportion of nonfatal OD cases leads to intensive care unit (ICU) admission, little is known about the risk factors of OD for ICU admissions. [24]. This is essential to understand the severity of the opioid epidemic and anticipate critical care needs for patients with OD. There has been inadequate work on assessing risk factors associated with OD leading to ICU admission, which may be important in comprehensively preventing the public health problem of ODs.

In this study, we specifically focused on the ICU setting to address the aforementioned issues. To mitigate the scarcity of structured SBDH information, we used an NLP system to automatically extract SBDH information from EHR notes and integrated that with available structured SBDH data entered upon admission. Then, we investigated the associations of various demographic, SBDH, and clinical variables with nonfatal OD for eligible ICU admissions. To date, none of the studies on OD utilized the EHR text for extracting SBDH information and few focused on the ICU setting. We bridge this gap by (1) showing that NLP systems can help extract SBDH information when structured data are inadequate and (2) identifying the risk factors that are crucial to the characterization of nonfatal OD leading to ICU admission.

## Methods

### Dataset

**Figure 1:**
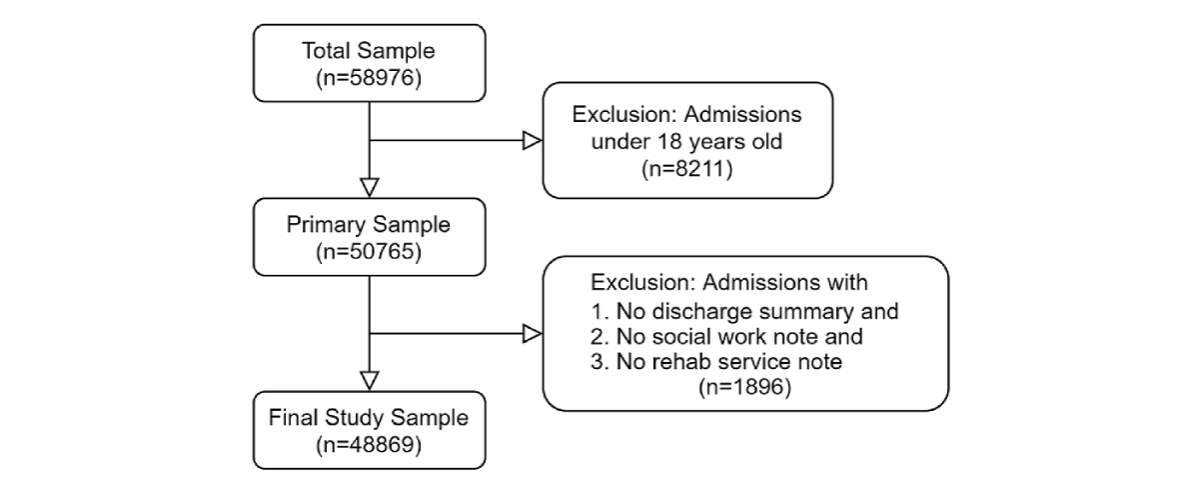
Data selection process.

Our primary data source is MIMIC-III [25], one of the largest publicly available ICU databases encompassing 12 years of data (2001-2012) from Beth Israel Deaconess Medical Center. First, we excluded admission data from patients who were less than 18 years old at the time of admission. For inclusion, admissions were also required to have at least one note from any of these 3 categories: discharge summary, social work note, or rehabilitation service note. We selected these 3 types of notes to maximize the use of social and behavioral information for SBDH extraction: Discharge summaries are a comprehensive summary of a patient’s hospital stay, social work notes focus specifically on the social nature of a patient’s life, and rehabilitation service notes focus on improving patients’ function and mobility to stabilize them for discharge. The final sample consisted of 48,869 hospital admissions from 37,361 patients. An overview of the data selection process is shown in Figure 1.

### Variables

All baseline variables were grouped into 3 categories: demographic, clinical, and SBDH. The demographic variables included age (18-39 years, 40-64 years, >64 years), gender (male or female), race/ethnicity (White, Black, Hispanic, or others), and marital status (married, divorced, widowed, single, or unknown marital status). As clinical variables, we considered drug use disorder, bipolar disorder, tobacco use disorder, major depressive disorder, alcohol use disorder, cirrhosis, chronic obstructive pulmonary disease (COPD), and renal insufficiency. This comprehensive list was made based on earlier studies related to OD [26-29], clinical judgment, and statistical analyses (see the “Statistical Analysis” section for further details). All clinical variables were detected using the International Classification of Disease, 9th Revision (ICD-9) codes from the admission diagnosis chart and included as dichotomous variables. The list of ICD-9 codes is available in Multimedia Appendix 1.

For SBDH variables, we used NLP to analyze the unstructured text data available in MIMIC-III. For each type of note, we chose the most relevant sections to extract the SBDH information: (1) discharge summaries: “Social History” sections; (2) social worker notes: “Patient/Family Assessment,” “Past Addictions History,” “Past Medical History” sections; (3) rehabilitation services: “Sexual and Social History” section.

We used the popular clinical NLP tool medSpaCy [30] to extract these sections from a note. We randomly chose a note, extracted the relevant sections as mentioned, and annotated for 6 categories of SBDH information. This process was repeatedly followed until we reached 1000 notes with at least one SBDH annotation. This annotated subset was later used to train a Bidirectional Encoder Representations from Transformers (BERT) model to extract SBDH at the word level. BERT [31] is a state-of-the-art language representation model that has successfully outperformed many other NLP systems across a wide range of tasks. We used the trained model to predict the SBDH information for the remaining notes. For an admission with multiple notes of the same type, we took the last note as representative of that admission as it typically includes the content of all the previous notes.

The 6 SBDH variables we chose were (1) housing insecurity, (2) unemployment, (3) social isolation, (4) alcohol use, (5) tobacco use, and (6) illicit drug use. The first 3 are social determinants and were selected based on the list of well-accepted social determinants provided by the Kaiser Family Foundation [32]. The rest were substance use–related health risk behaviors (ie, behavioral determinants) that were chosen for their clinical significance and relevance to OD. Details about the annotation process, NLP model development, and SBDH variable extraction procedures are provided in Multimedia Appendix 2.

In addition to the NLP-derived SBDH variables, we also identified social determinants from the structured data. We used the ICD-9 codes from patient diagnoses [33] to construct these 3 SBDH variables: (1) housing insecurity, (2) unemployment, and (3) social isolation. These were later integrated with the NLP-derived SBDH variables and prioritized in case of any mismatch. For example, if the NLP system detected “housing insecurity” as “No” for an admission and we obtained “Yes” from that admission’s diagnoses codes, we considered “Yes” as the correct value. In the end, there were 41,669 admissions (41,669/48,869, 85.27%) with at least one SBDH variable. Table 1 illustrates the 6 SBDH variables with brief descriptions and examples. If an admission had no mention of SBDH information, SBDH variables were coded as “unknown.” For instance, if an admission had no mention of patient housing status in the corresponding notes, homelessness was considered “unknown.” Other than these 3 SBDH variables, we also extracted insurance provider (private, Medicaid, Medicare, other government, or self-pay) information using ICD-9 codes.

**Table 1:** Descriptions and examples of social and behavioral determinants of health (SBDH) variables.

SBDH Variable		Description and example
**Housing insecurity**		
	Yes	Lack housing or stable shelter. Example: *homeless*, living with friends.
	No	Has access to housing. Example: *lives* in [**location**] by himself.
**Unemployment**		
	Yes	Patient has no source of income or lost job. Example: Patient used to work for the state lottery system, currently *unemployed*.
	No	Patient has employment or some source of income. Example: He works for [**Company**].
**Social isolation**		
	Yes	Lack of social support or community engagement. Example: Lives *alone* in [**Location**].
	No	Presence of social support. Example: He is *married* and *lives* with his wife.
**Alcohol use**		
	Current	Patient currently consumes alcohol. Example: two glasses of *wine* per night and 3 bottles over the weekend.
	Former	Patient has a history of alcohol consumption. Example: He has a history of *alcohol* abuse.
	None	Patient never consumed alcohol. Example: She denies any *alcohol use*.
**Smoking**		
	Current	Patient currently smokes. Example: He *smokes one pack of cigarettes* per week.
	Former	Patient has a history of tobacco usage. Example: The patient has a past history of *smoking*.
	None	Patient never consumed tobacco. Example: She is a *nonsmoker*.
**Illicit drug use**		
	Current	Patient uses non-prescribed controlled substance. Example: occasional *marijuana* use.
	Former	Patient has a history of using non-prescribed controlled substance. Example: Has a h/o^a^ of *cocaine* and *marijuana* abuse.
	None	Patient never used non-prescribed controlled substance, e.g., cocaine, marijuana. Example: Does not drink alcohol or use recreational *drugs*.

^a^h/o: history of.

### Outcome

The outcome was nonfatal OD, which was identified using ICD-9 codes [34].

### Statistical Analysis

First, we performed correlation and collinearity analyses for all the variables. The correlation plot and variance inflation factor [35] did not show multicollinearity among the variables. For the clinical variables, based on earlier work and task relevance, we chose 14 comorbidities: posttraumatic stress disorder, major depressive disorder, bipolar disorder, schizophrenia, alcohol use disorder, drug use disorder, tobacco use disorder, hepatitis C, diabetes, congestive heart failure, obstructive sleep apnea, COPD, cirrhosis, and renal insufficiency. We built logistic regression models and employed the sequential forward selection procedure [36] to identify the most essential clinical variables related to OD. The final list included 8 clinical variables: drug use disorder, bipolar disorder, tobacco use disorder, major depressive disorder, alcohol use disorder, cirrhosis, COPD, and renal insufficiency.

We used a logistic regression model to examine the associations of nonfatal OD with demographic, SBDH, and clinical variables. This was assessed in terms of adjusted odds ratios (aOR) with 95% CIs. We also evaluated the crude odds ratio (OR) with 95% CIs. The statistical significance was measured at *P*<.05. Hosmer-Lemeshow test was conducted and indicated a sufficient fit for our model (χ_8_=10.39; *P*=.24). All statistical analyses in this study were conducted in R (version 4.0.2).

## Results

### Descriptive Analysis

Table 2 presents the characteristics of our cohort (n=48,869). Our sample was comprised of mostly men (27,436/48,869, 56.14%) and white (35,058/48,869, 71.74%) adults. The majority of patients were aged 64 years or older (25,276/48,869, 51.72%). Of the clinical variables, renal insufficiency was the most prevalent (8158/48,869, 16.69%), followed by COPD (5674/48,869, 11.61%) and alcohol use disorder (4121/48,869, 8.43%). In our cohort, we observed that 7.28% (3559/48,869) of the patients were unemployed, 13.35% (6523/48,869) were socially isolated, and 0.82% (402/48,869) had housing insecurity. We found 171 (171/48,869, 0.35%) admissions with nonfatal OD.

**Table 2:** Prevalence of demographic, clinical, and social and behavioral determinants of health (SBDH) variables in MIMIC-III.

Variables			Overall (n=48,869)		With OD^a^ (n=171)		Without OD (n=48,698)
**Age^b^ (years), n (%)**							
	<40	4715 (9.65)		62 (36.26)		4653 (9.55)	
	40-64	18,878 (38.63)		92 (53.80)		18,786 (38.58)	
	>64	25,276 (51.72)		17 (9.94)		25,259 (51.87)	
**Gender,^b^ n (%)**							
	Male	27,436 (56.14)		100 (58.48)		27,336 (56.13)	
	Female	21,433 (43.86)		71 (41.52)		21,362 (43.87)	
**Race/ethnicity,^b^ n (%)**							
	White	35,058 (71.74)		127 (74.27)		34,931 (71.73)	
	Black	4694 (9.61)		13 (7.60)		4681 (9.61)	
	Hispanic	1664 (3.40)		8 (4.68)		1656 (3.40)	
	Other	7453 (15.25)		23 (13.45)		7430 (15.26)	
**Marital status,^b^ n (%)**							
	Married	23,378 (47.84)		42 (24.56)		23,336 (47.92)	
	Divorced	3664 (7.50)		22 (12.87)		3642 (7.48)	
	Widowed	7018 (14.36)		6 (3.51)		7012 (14.40)	
	Single	12,329 (25.23)		78 (45.61)		12,251 (25.16)	
	Unknown	2480 (5.07)		23 (13.45)		2457 (5.04)	
**Clinical variables,^b^ n (%)**							
	Drug use disorder	1493 (3.06)		80 (46.78)		1413 (2.90)	
	Bipolar disorder	1009 (2.06)		28 (16.37)		981 (2.01)	
	Tobacco use disorder	3274 (6.70)		20 (11.70)		3254 (6.68)	
	Major depressive disorder	298 (0.61)		7 (4.09)		291 (0.60)	
	Alcohol use disorder	4121 (8.43)		37 (21.64)		4084 (8.39)	
	Cirrhosis	2431 (4.97)		19 (11.11)		2412 (4.95)	
	COPD^c^	5674 (11.61)		18 (10.53)		5656 (11.61)	
	Renal insufficiency	8158 (16.69)		12 (7.02)		8146 (16.73)	
**Social determinant^d^: insurance provider, n (%)**							
	Private	15,371 (31.45)		43 (25.15)		15,328 (31.48)	
	Medicaid	4307 (8.81)		60 (35.09)		4247 (8.72)	
	Medicare	27,365 (56.00)		48 (28.07)		27,317 (56.09)	
	Government (others)	1324 (2.71)		14 (8.19)		1310 (2.69)	
	Self-pay	502 (1.03)		6 (3.50)		496 (1.02)	
**Social determinant^d^: housing insecurity, n (%)**							
	Yes	402 (0.82)		10 (5.85)		392 (0.80)	
	No	27,119 (55.49)		92 (53.80)		27,027 (55.50)	
	Unknown	21,348 (43.69)		69 (40.35)		21,279 (43.70)	
**Social determinant^d^: unemployment, n (%)**							
	Yes	3559 (7.28)		37 (21.64)		3522 (7.22)	
	No	12,671 (25.93)		31 (18.13)		12,640 (25.96)	
	Unknown	32,639 (66.79)		103 (60.23)		32,536 (66.82)	
**Social determinant^d^: social isolation, n (%)**							
	Yes	6523 (13.35)		23 (13.45)		6500 (13.35)	
	No	24,001 (49.11)		86 (50.29)		23,915 (49.11)	
	Unknown	18,345 (37.54)		62 (36.26)		18,283 (37.54)	
**Substance use^e^: alcohol use, n (%)**							
	Current	14,150 (28.96)		70 (40.94)		14,080 (28.91)	
	Former	2333 (4.77)		9 (5.26)		2324 (4.77)	
	None	15,378 (31.47)		40 (23.39)		15,338 (31.50)	
	Unknown	17,008 (34.80)		52 (30.41)		16,956 (34.82)	
**Substance use^e^: smoking, n (%)**							
	Current	6954 (14.23)		62 (36.26)		6892 (14.15)	
	Former	12,032 (24.62)		23 (13.45)		12,009 (24.66)	
	None	13,963 (28.57)		30 (17.54)		13,933 (28.61)	
	Unknown	15,920 (32.58)		56 (32.75)		15,864 (32.58)	
**Substance use^e^: illicit drug use, n (%)**							
	Current	1796 (3.67)		49 (28.65)		1747 (3.59)	
	Former	1362 (2.79)		26 (15.20)		1336 (2.74)	
	None	13,908 (28.46)		31 (18.13)		13,877 (28.50)	
	Unknown	31,803 (65.08)		65 (38.02)		31,738 (65.17)	

^a^OD: opioid overdose.

^b^Variables extracted from structured data.

^c^COPD: chronic obstructive pulmonary disease.

^d^Variables extracted from only structured data (insurance provider) or both structured data and unstructured text notes (natural language processing [NLP]).

^e^Variables extracted from unstructured text notes (NLP).

Of the 6 NLP-derived SBDH variables, only housing insecurity, unemployment, and social isolation had associated ICD-9 diagnostic codes. Compared with their NLP-derived counterparts, these structured variables were coded infrequently. For example, using ICD-9 codes, we found 258 admissions with “housing insecurity,” whereas the NLP system detected 402 admissions. For “unemployment,” it was 20 for the ICD-9 codes and 10,876 for the NLP system. And more striking, for “social isolation,” only 4 admissions had relevant ICD-9 codes in their diagnosis compared to 6523 admissions found by the NLP system. Due to the substantial prevalence gap, we did not compare the quality of these 2 types of SBDH variables side by side. In all, structured SBDH variables accounted for only 0.18% of the SBDH variables. This clearly shows that NLP can be useful to extract SBDH information from EHR notes when structured data are not enough. This also helps reduce bias from the use of structured data only.

### Multivariable Logistic Regression Analysis

Several factors were strongly associated with nonfatal OD (Table 3). Among the demographic risk factors, Blacks (aOR 0.51, 95% CI 0.28-0.94) and older age groups (40-64 years: aOR 0.65, 95% CI 0.44-0.96; >64 years: aOR 0.16, 95% CI 0.08-0.34) had lower odds compared with White and younger patients. Among the 8 clinical variables, 5 were strong risk factors for nonfatal OD. We observed increased odds of overdose among individuals with drug use disorder (aOR 8.17, 95% CI 5.44-12.27), bipolar disorder (aOR 2.69, 95% CI 1.68-4.29), and major depressive disorder (aOR 2.57, 95% CI 1.12-5.88). Interestingly, tobacco use disorder (aOR 0.53, 95% CI 0.32-0.89) and alcohol use disorder (aOR 0.64, 95% CI 0.42-1.00) had decreased odds. Among the SBDH variables, individuals with Medicaid had increased odds compared with those with private medical insurance (aOR 2.26, 95% CI 1.43-3.58). History of (aOR 2.09, 95% CI 1.15-3.79) and current (aOR 2.06, 95% CI 1.20-3.55) use of illicit drugs were also strongly associated with the outcome.

**Table 3:** Multivariable logistic regression analysis for the factors associated with nonfatal opioid overdose (OD).

Variables		Crude OR^a^	95% CI	aOR^b^	95% CI
**Age (years)**					
	<40	Ref^c^	Ref	Ref	Ref
	40-64	0.37	0.27-0.51	0.65	0.44-0.96
	>64	0.05	0.03-0.08	0.16	0.08-0.34
**Gender**					
	Male	Ref	Ref	Ref	Ref
	Female	0.91	0.67-1.23	1.13	0.81-1.58
**Race/ethnicity**					
	White	Ref	Ref	Ref	Ref
	Black	0.76	0.41-1.30	0.51	0.28-0.94
	Hispanic	1.33	0.60-2.55	0.69	0.33-1.45
	Others	0.85	0.53-1.30	0.59	0.35-0.98
**Marital status**					
	Married	Ref	Ref	Ref	Ref
	Divorced	3.36	1.97-5.57	1.56	0.89-2.74
	Widowed	0.48	0.18-1.04	0.76	0.30-1.88
	Single	3.54	2.44-5.19	1.03	0.65-1.61
	Unknown	5.20	3.08-8.58	2.85	1.55-5.24
**Clinical variables**					
	Drug use disorder	29.42	21.65-39.90	8.17	5.44-12.27
	Bipolar disorder	9.52	6.20-14.12	2.69	1.68-4.29
	Tobacco use disorder	1.85	1.12-2.88	0.53	0.32-0.89
	Major depressive disorder	7.10	2.99-14.16	2.57	1.12-5.88
	Alcohol use disorder	3.02	2.06-4.30	0.64	0.42-1.00
	Cirrhosis	2.40	1.44-3.77	1.65	0.97-2.82
	COPD^d^	7.10	2.99-14.16	1.65	0.97-2.81
	Renal insufficiency	3.02	2.06-4.30	0.62	0.33-1.15
**Social determinant: insurance type**					
	Private	Ref	Ref	Ref	Ref
	Medicaid	5.04	3.41-7.50	2.26	1.43-3.58
	Medicare	0.63	0.41-0.95	1.34	0.81-2.23
	Government (others)	3.81	2.01-6.80	1.90	0.99-3.65
	Self-paid	4.31	1.64-9.42	1.83	0.73-4.56
**Social determinant: housing insecurity**					
	No	Ref	Ref	Ref	Ref
	Yes	7.49	3.63-13.80	0.98	0.47-2.06
	Unknown	0.95	0.69-1.30	0.89	0.60-1.33
**Social determinant: unemployment**					
	No	Ref	Ref	Ref	Ref
	Yes	4.28	2.66-6.95	1.10	0.65-1.87
	Unknown	1.29	0.87-1.96	0.73	0.47-1.14
**Social determinant: social isolation**					
	No	Ref	Ref	Ref	Ref
	Yes	0.98	0.61-1.53	0.97	0.59-1.60
	Unknown	0.94	0.68-1.31	1.01	0.66-1.53
**Substance use: alcohol use**					
	None	Ref	Ref	Ref	Ref
	Former	1.48	0.67-2.92	0.66	0.30-1.44
	Current	1.91	1.30-2.84	1.11	0.71-1.72
	Unknown	1.18	0.78-1.79	1.05	0.62-1.78
**Substance use: smoking**					
	None	Ref	Ref	Ref	Ref
	Former	0.89	0.51-1.53	0.92	0.52-1.65
	Current	4.18	2.72-6.55	1.40	0.84-2.33
	Unknown	1.64	1.06-2.59	1.12	0.64-1.96
**Substance use: illicit drug use**					
	None	Ref	Ref	Ref	Ref
	Former	8.71	5.12-14.7	2.09	1.15-3.79
	Current	12.56	8.03-19.93	2.06	1.20-3.55
	Unknown	0.92	0.60-1.42	1.05	0.65-1.70

^a^OR: odds ratio.

^b^aOR: adjusted odds ratio.

^c^Ref: Reference.

^d^COPD: chronic obstructive pulmonary disorder.

## Discussion

### Principal Findings

To our knowledge, this is the first study to examine the risk factors associated with nonfatal OD leading to ICU admission. In the United States, the need for characterizing critical care patients with OD is rising [23,24], and this study partially addressed that by identifying the risk factors for nonfatal OD from a large ICU database. The novelty also lies in the use of a state-of-the-art NLP system that utilized unstructured EHR notes for essential SBDH extraction due to inadequate representation from structured data. There is a growing body of literature showing that SBDH can strongly influence patient health and outcomes [12]. For example, SBDH variables have been shown to be strongly associated with suicide attempt [11], mortality [17], and mental health diagnosis [18]. The challenges here for the health care systems are to set up methods that can identify SBDH and use them at the point of care to inform clinical action [37,38]. Our work demonstrated that using NLP to detect SBDH information from EHR text can be a viable option in this regard.

According to our analysis, multiple SBDH variables were significantly associated with nonfatal OD in ICU settings. We observed that patients with economic instability (unemployed) were more likely to have an overdose, but homelessness and social isolation conferred little additional risk. Among behavioral determinants, *current* alcohol users and smokers had higher odds of overdose, whereas *former* users had decreased odds. Illicit drug use was strongly associated with nonfatal OD for both *former* and *current* users. Among clinical variables, tobacco use disorder and alcohol use disorder had strong negative associations with nonfatal OD. We hypothesize that the majority of the patients diagnosed with such disorders were already receiving additional social counseling or clinical support, which helped them build better health and behavioral practices. However, we did not have enough relevant admission data in MIMIC-III to validate this hypothesis; future research is needed to identify the reasons for this observation.

### Limitations

There are several limitations of our study. EHR data are prone to variability by provider documentation and may contain incomplete SBDH information [39]. Additionally, using only ICD-9 codes to identify different medical conditions may lead to inaccurate or misleading values for the corresponding variable. However, structured data often significantly lack SBDH information (only 0.18% for this study), making an NLP-based approach a valuable integration for population studies. Finally, our data had a very low prevalence of nonfatal OD cases (171/48,869, 0.35%), and the MIMIC (ICU) database might not characterize the general outpatient/inpatient hospital setting.

While our study describes an important methodological process that can identify important SBDH factors to consider, which is a necessary first step, further research is needed on subsequent steps on how best to share and translate this information to providers so that they can effectively and actionably use the findings. As our future work, we would like to work on modeling the NLP system predictions for SBDH extraction and how they can be better tied with predictor assessment metrics (eg, OR).

### Conclusions

This is the first work to evaluate the risk factors associated with nonfatal OD leading to ICU admissions. Our work concluded that data-driven NLP systems can be largely beneficial in the automatic extraction of SBDH information from unstructured EHR text data. We also showed that analyzing critical care admissions is crucial to better understand the opioid epidemic. Utilizing NLP to leverage the rich EHR notes and more epidemiological studies in critical care settings could be useful for deeper analysis of the OD crisis, leading to the development of better risk assessment tools and effective prevention systems.

## Appendix

Multimedia Appendix 1 International Classification of Disease, 9th Revision codes for clinical variables.

Multimedia Appendix 2 Natural language processing model training and evaluation.

## Abbreviations

aOR: adjusted odds ratio
BERT: Bidirectional Encoder Representations from Transformers
COPD: chronic obstructive pulmonary disease
EHR: Electronic health record
ICD-9: International Classification of Disease, 9th Revision
ICU: Intensive care unit
NIH: National Institutes of Health
NLP: natural language processing
OD: opioid overdose
OR: odds ratio
SBDH: social and behavioral determinants of health
SDOH: social determinants of health
