# Supplementary material for: Risk Factors Associated With Nonfatal Opioid Overdose Leading to Intensive Care Unit Admission: A Cross-sectional Study
Source: JMIR Med Inform. 2021 Nov 8;9(11):e32851. doi: 10.2196/32851 (PMC8663596; doi:10.2196/32851)
Supplement: Multimedia Appendix 1 [file medinform_v9i11e32851_app1.docx]

## Multimedia Appendix 1: ICD-9 codes for clinical variables

### Drug Use Disorder

| ICD-9 code | Description |
| --- | --- |
| 304 | Opioid type dependence, unspecified |
| 304.01 | Opioid type dependence, continuous |
| 304.02 | Opioid type dependence, episodic |
| 304.03 | Opioid type dependence, in remission |
| 304.1 | Sedative, hypnotic or anxiolytic dependence, unspecified |
| 304.11 | Sedative, hypnotic or anxiolytic dependence, continuous |
| 304.12 | Sedative, hypnotic or anxiolytic dependence, episodic |
| 304.13 | Sedative, hypnotic or anxiolytic dependence, in remission |
| 304.2 | Cocaine dependence, unspecified |
| 304.21 | Cocaine dependence, continuous |
| 304.22 | Cocaine dependence, episodic |
| 304.23 | Cocaine dependence, in remission |
| 304.3 | Cannabis dependence, unspecified |
| 304.31 | Cannabis dependence, continuous |
| 304.32 | Cannabis dependence, episodic |
| 304.33 | Cannabis dependence, in remission |
| 304.4 | Amphetamine and other psychostimulant dependence, unspecified |
| 304.41 | Amphetamine and other psychostimulant dependence, continuous |
| 304.42 | Amphetamine and other psychostimulant dependence, episodic |
| 304.43 | Amphetamine and other psychostimulant dependence, in remission |
| 304.5 | Hallucinogen dependence, unspecified |
| 304.51 | Hallucinogen dependence, continuous |
| 304.52 | Hallucinogen dependence, episodic |
| 304.53 | Hallucinogen dependence, in remission |
| 304.6 | Other specified drug dependence, unspecified |
| 304.61 | Other specified drug dependence, continuous |
| 304.62 | Other specified drug dependence, episodic |
| 304.63 | Other specified drug dependence, in remission |
| 304.7 | Combinations of opioid type drug with any other drug dependence, unspecified |
| 304.71 | Combinations of opioid type drug with any other drug dependence, continuous |
| 304.72 | Combinations of opioid type drug with any other drug dependence, episodic |
| 304.73 | Combinations of opioid type drug with any other drug dependence, in remission |
| 304.8 | Combinations of drug dependence excluding opioid type drug, unspecified |
| 304.81 | Combinations of drug dependence excluding opioid type drug, continuous |
| 304.82 | Combinations of drug dependence excluding opioid type drug, episodic |
| 304.83 | Combinations of drug dependence excluding opioid type drug, in remission |
| 304.9 | Unspecified drug dependence, unspecified |
| 304.91 | Unspecified drug dependence, continuous |
| 304.92 | Unspecified drug dependence, episodic |
| 304.93 | Unspecified drug dependence, in remission |
| 305.2 | Cannabis abuse, unspecified |
| 305.21 | Cannabis abuse, continuous |
| 305.22 | Cannabis abuse, episodic |
| 305.23 | Cannabis abuse, in remission |
| 305.3 | Hallucinogen abuse, unspecified |
| 305.31 | Hallucinogen abuse, continuous |
| 305.32 | Hallucinogen abuse, episodic |
| 305.33 | Hallucinogen abuse, in remission |
| 305.4 | Sedative, hypnotic or anxiolytic abuse, unspecified |
| 305.41 | Sedative, hypnotic or anxiolytic abuse, continuous |
| 305.42 | Sedative, hypnotic or anxiolytic abuse, episodic |
| 305.43 | Sedative, hypnotic or anxiolytic abuse, in remission |
| 305.5 | Opioid abuse, unspecified |
| 305.51 | Opioid abuse, continuous |
| 305.52 | Opioid abuse, episodic |
| 305.53 | Opioid abuse, in remission |
| 305.6 | Cocaine abuse, unspecified |
| 305.61 | Cocaine abuse, continuous |
| 305.62 | Cocaine abuse, episodic |
| 305.63 | Cocaine abuse, in remission |
| 305.7 | Amphetamine or related acting sympathomimetic abuse, unspecified |
| 305.71 | Amphetamine or related acting sympathomimetic abuse, continuous |
| 305.72 | Amphetamine or related acting sympathomimetic abuse, episodic |
| 305.73 | Amphetamine or related acting sympathomimetic abuse, in remission |
| 305.8 | Antidepressant type abuse, unspecified |
| 305.81 | Antidepressant type abuse, continuous |
| 305.82 | Antidepressant type abuse, episodic |
| 305.83 | Antidepressant type abuse, in remission |
| 292 | Drug withdrawal |
| 292.11 | Drug-induced psychotic disorder with delusions |
| 292.12 | Drug-induced psychotic disorder with hallucinations |

### Bipolar Disorder

| ICD-9 code | Description |
| --- | --- |
| 296.40 | Bipolar I disorder, most recent episode (or current) manic, unspecified |
| 296.41 | Bipolar I disorder, most recent episode (or current) manic, mild |
| 296.42 | Bipolar I disorder, most recent episode (or current) manic, moderate |
| 296.43 | Bipolar I disorder, most recent episode (or current) manic, severe, without mention of psychotic behavior |
| 296.44 | Bipolar I disorder, most recent episode (or current) manic, severe, specified as with psychotic behavior |
| 296.45 | Bipolar I disorder, most recent episode (or current) manic, in partial or unspecified remission |
| 296.46 | Bipolar I disorder, most recent episode (or current) manic, in full remission |
| 296.50 | Bipolar I disorder, most recent episode (or current) depressed, unspecified |
| 296.51 | Bipolar I disorder, most recent episode (or current) depressed, mild |
| 296.52 | Bipolar I disorder, most recent episode (or current) depressed, moderate |
| 296.53 | Bipolar I disorder, most recent episode (or current) depressed, severe, without mention of psychotic behavior |
| 296.54 | Bipolar I disorder, most recent episode (or current) depressed, severe, specified as with psychotic behavior |
| 296.55 | Bipolar I disorder, most recent episode (or current) depressed, in partial or unspecified remission |
| 296.56 | Bipolar I disorder, most recent episode (or current) depressed, in full remission |
| 296.60 | Bipolar I disorder, most recent episode (or current) mixed, unspecified |
| 296.61 | Bipolar I disorder, most recent episode (or current) mixed, mild |
| 296.62 | Bipolar I disorder, most recent episode (or current) mixed, moderate |
| 296.63 | Bipolar I disorder, most recent episode (or current) mixed, severe, without mention of psychotic behavior |
| 296.64 | Bipolar I disorder, most recent episode (or current) mixed, severe, specified as with psychotic behavior |
| 296.65 | Bipolar I disorder, most recent episode (or current) mixed, in partial or unspecified remission |
| 296.66 | Bipolar I disorder, most recent episode (or current) mixed, in full remission |
| 296.7 | Bipolar I disorder, most recent episode (or current) unspecified |
| 296.80 | Bipolar disorder, unspecified |
| 296.81 | Atypical manic disorder |
| 296.82 | Atypical depressive disorder |
| 296.89 | Other bipolar disorders |
| 296.90 | Unspecified episodic mood disorder |
| 296.99 | Other specified episodic mood disorder |
| 296.00 | Bipolar I disorder, single manic episode, unspecified |
| 296.01 | Bipolar I disorder, single manic episode, mild |
| 296.02 | Bipolar I disorder, single manic episode, moderate |
| 296.03 | Bipolar I disorder, single manic episode, severe, without mention of psychotic behavior |
| 296.04 | Bipolar I disorder, single manic episode, severe, specified as with psychotic behavior |
| 296.05 | Bipolar I disorder, single manic episode, in partial or unspecified remission |
| 296.06 | Bipolar I disorder, single manic episode, in full remission |
| 296.10 | Manic affective disorder, recurrent episode, unspecified |
| 296.11 | Manic affective disorder, recurrent episode, mild |
| 296.12 | Manic affective disorder, recurrent episode, moderate |
| 296.13 | Manic affective disorder, recurrent episode, severe, without mention of psychotic behavior |
| 296.14 | Manic affective disorder, recurrent episode, severe, specified as with psychotic behavior |
| 296.15 | Manic affective disorder, recurrent episode, in partial or unspecified remission |
| 296.16 | Manic affective disorder, recurrent episode, in full remission |
| V11.1 | Personal history of affective disorders |

### Tobacco Use Disorder

| ICD-9 code | Description |
| --- | --- |
| 305.1 | Tobacco use disorder |

### Major Depressive Disorder

| ICD-9 code | Description |
| --- | --- |
| 296.34 | Major depressive affective disorder, recurrent episode, severe, specified as with psychotic behavior |
| 296.35 | Major depressive affective disorder, recurrent episode, in partial or unspecified remission |
| 296.36 | Major depressive affective disorder, recurrent episode, in full remission |
| 296.2 | Major depressive affective disorder, single episode, unspecified |
| 296.21 | Major depressive affective disorder, single episode, mild |
| 296.22 | Major depressive affective disorder, single episode, moderate |
| 296.23 | Major depressive affective disorder, single episode, severe, without mention of psychotic behavior |
| 296.24 | Major depressive affective disorder, single episode, severe, specified as with psychotic behavior |
| 296.25 | Major depressive affective disorder, single episode, in partial or unspecified remission |
| 296.26 | Major depressive affective disorder, single episode, in full remission |
| 296.3 | Major depressive affective disorder, recurrent episode, unspecified |
| 296.31 | Major depressive affective disorder, recurrent episode, mild |
| 296.32 | Major depressive affective disorder, recurrent episode, moderate |
| 296.33 | Major depressive affective disorder, recurrent episode, severe, without mention of psychotic behavior |

### Alcohol Use Disorder

| ICD-9 code | Description |
| --- | --- |
| 303.00 | Acute alcoholic intoxication in alcoholism, unspecified |
| 303.01 | Acute alcoholic intoxication in alcoholism, continuous |
| 303.02 | Acute alcoholic intoxication in alcoholism, episodic |
| 303.03 | Acute alcoholic intoxication in alcoholism, in remission |
| 303.90 | Other and unspecified alcohol dependence, unspecified |
| 303.91 | Other and unspecified alcohol dependence, continuous |
| 303.92 | Other and unspecified alcohol dependence, episodic |
| 303.93 | Other and unspecified alcohol dependence, in remission |
| 305.00 | Alcohol abuse, unspecified |
| 305.01 | Alcohol abuse, continuous |
| 305.02 | Alcohol abuse, episodic |
| 305.03 | Alcohol abuse, in remission |
| 291.0 | Alcohol withdrawal delirium |
| 291.1 | Alcohol-induced persisting amnestic disorder |
| 291.2 | Alcohol-induced persisting dementia |
| 291.3 | Alcohol-induced psychotic disorder with hallucinations |
| 291.4 | Idiosyncratic alcohol intoxication |
| 291.5 | Alcohol-induced psychotic disorder with delusions |
| 291.81 | Alcohol withdrawal |
| 291.82 | Alcohol induced sleep disorders |
| 291.89 | Other alcohol-induced mental disorders |
| 291.9 | Unspecified alcohol-induced mental disorders |
| 357.5 | Alcoholic polyneuropathy |
| 425.5 | Alcoholic cardiomyopathy |
| 571.0 | Alcoholic fatty liver |
| 571.1 | Acute alcoholic hepatitis |
| 571.2 | Alcoholic cirrhosis of liver |
| 571.3 | Alcoholic liver damage, unspecified |
| 535.30 | Alcoholic gastritis, without mention of hemorrhage |
| V11.3 | Personal history of alcoholism |

### Cirrhosis

| ICD-9 code | Description |
| --- | --- |
| 571.2 | Alcoholic cirrhosis of liver |
| 571.5 | Cirrhosis of liver without mention of alcohol |
| 571.6 | Biliary cirrhosis |

### Chronic obstructive pulmonary disease (COPD)

| ICD-9 code | Description |
| --- | --- |
| 490 | Bronchitis, not specified as acute or chronic |
| 491 | Simple chronic bronchitis |
| 491.1 | Mucopurulent chronic bronchitis |
| 491.2 | Obstructive chronic bronchitis without exacerbation |
| 491.21 | Obstructive chronic bronchitis with (acute) exacerbation |
| 491.22 | Obstructive chronic bronchitis with acute bronchitis |
| 491.8 | Other chronic bronchitis |
| 491.9 | Unspecified chronic bronchitis |
| 496 | Chronic airway obstruction, not elsewhere classified |

### Renal Insufficiency

| ICD-9 code | Description |
| --- | --- |
| 403.01 | Hypertensive chronic kidney disease, malignant, with chronic kidney disease stage V or end stage renal disease |
| 403.11 | Hypertensive chronic kidney disease, benign, with chronic kidney disease stage V or end stage renal disease |
| 403.91 | Hypertensive chronic kidney disease, unspecified, with chronic kidney disease stage V or end stage renal disease |
| 404.02 | Hypertensive heart and chronic kidney disease, malignant, without heart failure and with chronic kidney disease stage V or end stage renal disease |
| 404.03 | Hypertensive heart and chronic kidney disease, malignant, with heart failure and with chronic kidney disease stage V or end stage renal disease |
| 404.12 | Hypertensive heart and chronic kidney disease, benign, without heart failure and with chronic kidney disease stage V or end stage renal disease |
| 404.13 | Hypertensive heart and chronic kidney disease, benign, with heart failure and chronic kidney disease stage V or end stage renal disease |
| 404.92 | Hypertensive heart and chronic kidney disease, unspecified, without heart failure and with chronic kidney disease stage V or end stage renal disease |
| 404.93 | Hypertensive heart and chronic kidney disease, unspecified, with heart failure and chronic kidney disease stage V or end stage renal disease |
| 581.0 | Nephrotic syndrome with lesion of proliferative glomerulonephritis |
| 581.1 | Nephrotic syndrome with lesion of membranous glomerulonephritis |
| 581.2 | Nephrotic syndrome with lesion of membranoproliferative glomerulonephritis |
| 581.3 | Nephrotic syndrome with lesion of minimal change glomerulonephritis |
| 581.81 | Nephrotic syndrome in diseases classified elsewhere |
| 581.89 | Nephrotic syndrome with other specified pathological lesion in kidney |
| 581.9 | Nephrotic syndrome with unspecified pathological lesion in kidney |
| 582.0 | Chronic glomerulonephritis with lesion of proliferative glomerulonephritis |
| 582.1 | Chronic glomerulonephritis with lesion of membranous glomerulonephritis |
| 582.2 | Chronic glomerulonephritis with lesion of membranoproliferative glomerulonephritis |
| 582.4 | Chronic glomerulonephritis with lesion of rapidly progressive glomerulonephritis |
| 582.81 | Chronic glomerulonephritis in diseases classified elsewhere |
| 582.89 | Chronic glomerulonephritis with other specified pathological lesion in kidney |
| 582.9 | Chronic glomerulonephritis with unspecified pathological lesion in kidney |
| 583.0 | Nephritis and nephropathy, not specified as acute or chronic, with lesion of proliferative glomerulonephritis |
| 583.1 | Nephritis and nephropathy, not specified as acute or chronic, with lesion of membranous glomerulonephritis |
| 583.2 | Nephritis and nephropathy, not specified as acute or chronic, with lesion of membranoproliferative glomerulonephritis |
| 583.4 | Nephritis and nephropathy, not specified as acute or chronic, with lesion of rapidly progressive glomerulonephritis |
| 583.6 | Nephritis and nephropathy, not specified as acute or chronic, with lesion of renal cortical necrosis |
| 583.7 | Nephritis and nephropathy, not specified as acute or chronic, with lesion of renal medullary necrosis |
| 583.81 | Nephritis and nephropathy, not specified as acute or chronic, in diseases classified elsewhere |
| 583.89 | Nephritis and nephropathy, not specified as acute or chronic, with other specified pathological lesion in kidney |
| 583.9 | Nephritis and nephropathy, not specified as acute or chronic, with unspecified pathological lesion in kidney |
| 585.1 | Chronic kidney disease, Stage I |
| 585.2 | Chronic kidney disease, Stage II (mild) |
| 585.3 | Chronic kidney disease, Stage III (moderate) |
| 585.4 | Chronic kidney disease, Stage IV (severe) |
| 585.5 | Chronic kidney disease, Stage V |
| 585.6 | End stage renal disease |
| 585.9 | Chronic kidney disease, unspecified |
| 586. | Renal failure, unspecified |
| 587. | Renal sclerosis, unspecified |
| 588.0 | Renal osteodystrophy |
| 588.1 | Nephrogenic diabetes insipidus |
| 588.81 | Secondary hyperparathyroidism (of renal origin) |
| 588.89 | Other specified disorders resulting from impaired renal function |
| 588.9 | Unspecified disorder resulting from impaired renal function |
| V42.0 | Kidney replaced by transplant |
| V45.11 | Renal dialysis status |
| V45.12 | Noncompliance with renal dialysis |
| 792.5 | Cloudy (hemodialysis) (peritoneal) dialysis effluent |

### Post-traumatic stress disorder (PTSD)

| ICD-9 code | Description |
| --- | --- |
| 309.81 | Posttraumatic stress disorder |

### Schizophrenia

| ICD-9 code | Description |
| --- | --- |
| 295.00 | Simple type schizophrenia, unspecified |
| 295.01 | Simple type schizophrenia, subchronic |
| 295.02 | Simple type schizophrenia, chronic |
| 295.03 | Simple type schizophrenia, subchronic with acute exacerbation |
| 295.04 | Simple type schizophrenia, chronic with acute exacerbation |
| 295.05 | Simple type schizophrenia, in remission |
| 295.10 | Disorganized type schizophrenia, unspecified |
| 295.11 | Disorganized type schizophrenia, subchronic |
| 295.12 | Disorganized type schizophrenia, chronic |
| 295.13 | Disorganized type schizophrenia, subchronic with acute exacerbation |
| 295.14 | Disorganized type schizophrenia, chronic with acute exacerbation |
| 295.15 | Disorganized type schizophrenia, in remission |
| 295.20 | Catatonic type schizophrenia, unspecified |
| 295.21 | Catatonic type schizophrenia, subchronic |
| 295.22 | Catatonic type schizophrenia, chronic |
| 295.23 | Catatonic type schizophrenia, subchronic with acute exacerbation |
| 295.24 | Catatonic type schizophrenia, chronic with acute exacerbation |
| 295.25 | Catatonic type schizophrenia, in remission |
| 295.30 | Paranoid type schizophrenia, unspecified |
| 295.31 | Paranoid type schizophrenia, subchronic |
| 295.32 | Paranoid type schizophrenia, chronic |
| 295.33 | Paranoid type schizophrenia, subchronic with acute exacerbation |
| 295.34 | Paranoid type schizophrenia, chronic with acute exacerbation |
| 295.35 | Paranoid type schizophrenia, in remission |
| 295.40 | Schizophreniform disorder, unspecified |
| 295.41 | Schizophreniform disorder, subchronic |
| 295.42 | Schizophreniform disorder, chronic |
| 295.43 | Schizophreniform disorder, subchronic with acute exacerbation |
| 295.44 | Schizophreniform disorder, chronic with acute exacerbation |
| 295.45 | Schizophreniform disorder, in remission |
| 295.50 | Latent schizophrenia, unspecified |
| 295.51 | Latent schizophrenia, subchronic |
| 295.52 | Latent schizophrenia, chronic |
| 295.54 | Latent schizophrenia, chronic with acute exacerbation |
| 295.55 | Latent schizophrenia, in remission |
| 295.60 | Schizophrenic disorders, residual type, unspecified |
| 295.61 | Schizophrenic disorders, residual type, subchronic |
| 295.62 | Schizophrenic disorders, residual type, chronic |
| 295.63 | Schizophrenic disorders, residual type, subchronic with acute exacerbation |
| 295.64 | Schizophrenic disorders, residual type, chronic with acute exacerbation |
| 295.80 | Other specified types of schizophrenia, unspecified |
| 295.81 | Other specified types of schizophrenia, subchronic |
| 295.82 | Other specified types of schizophrenia, chronic |
| 295.83 | Other specified types of schizophrenia, subchronic with acute exacerbation |
| 295.84 | Other specified types of schizophrenia, chronic with acute exacerbation |
| 295.85 | Other specified types of schizophrenia, in remission |
| 295.90 | Unspecified schizophrenia, unspecified |
| 295.91 | Unspecified schizophrenia, subchronic |
| 295.92 | Unspecified schizophrenia, chronic |
| 295.93 | Unspecified schizophrenia, subchronic with acute exacerbation |
| 295.94 | Unspecified schizophrenia, chronic with acute exacerbation |
| 295.95 | Unspecified schizophrenia, in remission |
| V11.0 | Personal history of schizophrenia |

### Hepatitis C

| ICD-9 code | Description |
| --- | --- |
| 070.41 | Acute hepatitis C with hepatic coma |
| 070.44 | Chronic hepatitis C with hepatic coma |
| 070.51 | Acute hepatitis C without mention of hepatic coma |
| 070.54 | Chronic hepatitis C without mention of hepatic coma |
| V02.62 | Hepatitis C carrier |

### Diabetes

| ICD-9 code | Description |
| --- | --- |
| 250 | Diabetes mellitus without mention of complication, type II or unspecified type, not stated as uncontrolled |
| 250.01 | Diabetes mellitus without mention of complication, type I [juvenile type], not stated as uncontrolled |
| 250.02 | Diabetes mellitus without mention of complication, type II or unspecified type, uncontrolled |
| 250.03 | Diabetes mellitus without mention of complication, type I [juvenile type], uncontrolled |
| 250.1 | Diabetes with ketoacidosis, type II or unspecified type, not stated as uncontrolled |
| 250.11 | Diabetes with ketoacidosis, type I [juvenile type], not stated as uncontrolled |
| 250.12 | Diabetes with ketoacidosis, type II or unspecified type, uncontrolled |
| 250.13 | Diabetes with ketoacidosis, type I [juvenile type], uncontrolled |
| 250.2 | Diabetes with hyperosmolarity, type II or unspecified type, not stated as uncontrolled |
| 250.21 | Diabetes with hyperosmolarity, type I [juvenile type], not stated as uncontrolled |
| 250.22 | Diabetes with hyperosmolarity, type II or unspecified type, uncontrolled |
| 250.23 | Diabetes with hyperosmolarity, type I [juvenile type], uncontrolled |
| 250.3 | Diabetes with other coma, type II or unspecified type, not stated as uncontrolled |
| 250.31 | Diabetes with other coma, type I [juvenile type], not stated as uncontrolled |
| 250.32 | Diabetes with other coma, type II or unspecified type, uncontrolled |
| 250.33 | Diabetes with other coma, type I [juvenile type], uncontrolled |
| 250.4 | Diabetes with renal manifestations, type II or unspecified type, not stated as uncontrolled |
| 250.41 | Diabetes with renal manifestations, type I [juvenile type], not stated as uncontrolled |
| 250.42 | Diabetes with renal manifestations, type II or unspecified type, uncontrolled |
| 250.43 | Diabetes with renal manifestations, type I [juvenile type], uncontrolled |
| 250.5 | Diabetes with ophthalmic manifestations, type II or unspecified type, not stated as uncontrolled |
| 250.51 | Diabetes with ophthalmic manifestations, type I [juvenile type], not stated as uncontrolled |
| 250.52 | Diabetes with ophthalmic manifestations, type II or unspecified type, uncontrolled |
| 250.53 | Diabetes with ophthalmic manifestations, type I [juvenile type], uncontrolled |
| 250.6 | Diabetes with neurological manifestations, type II or unspecified type, not stated as uncontrolled |
| 250.61 | Diabetes with neurological manifestations, type I [juvenile type], not stated as uncontrolled |
| 250.62 | Diabetes with neurological manifestations, type II or unspecified type, uncontrolled |
| 250.63 | Diabetes with neurological manifestations, type I [juvenile type], uncontrolled |
| 250.7 | Diabetes with peripheral circulatory disorders, type II or unspecified type, not stated as uncontrolled |
| 250.71 | Diabetes with peripheral circulatory disorders, type I [juvenile type], not stated as uncontrolled |
| 250.72 | Diabetes with peripheral circulatory disorders, type II or unspecified type, uncontrolled |
| 250.73 | Diabetes with peripheral circulatory disorders, type I [juvenile type], uncontrolled |
| 250.8 | Diabetes with other specified manifestations, type II or unspecified type, not stated as uncontrolled |
| 250.81 | Diabetes with other specified manifestations, type I [juvenile type], not stated as uncontrolled |
| 250.82 | Diabetes with other specified manifestations, type II or unspecified type, uncontrolled |
| 250.83 | Diabetes with other specified manifestations, type I [juvenile type], uncontrolled |
| 250.9 | Diabetes with unspecified complication, type II or unspecified type, not stated as uncontrolled |
| 250.91 | Diabetes with unspecified complication, type I [juvenile type], not stated as uncontrolled |
| 250.92 | Diabetes with unspecified complication, type II or unspecified type, uncontrolled |
| 250.93 | Diabetes with unspecified complication, type I [juvenile type], uncontrolled |
| 357.2 | Polyneuropathy in diabetes |

### Congestive Heart Failure (CHF)

| ICD-9 code | Description |
| --- | --- |
| 402.01 | Malignant hypertensive heart disease with heart failure |
| 402.11 | Benign hypertensive heart disease with heart failure |
| 402.91 | Unspecified hypertensive heart disease with heart failure |
| 404.01 | Hypertensive heart and chronic kidney disease, malignant, with heart failure and with chronic kidney disease stage I through stage IV, or unspecified |
| 404.03 | Hypertensive heart and chronic kidney disease, malignant, with heart failure and with chronic kidney disease stage V or end stage renal disease |
| 404.11 | Hypertensive heart and chronic kidney disease, benign, with heart failure and with chronic kidney disease stage I through stage IV, or unspecified |
| 404.13 | Hypertensive heart and chronic kidney disease, benign, with heart failure and chronic kidney disease stage V or end stage renal disease |
| 404.91 | Hypertensive heart and chronic kidney disease, unspecified, with heart failure and with chronic kidney disease stage I through stage IV, or unspecified |
| 404.93 | Hypertensive heart and chronic kidney disease, unspecified, with heart failure and chronic kidney disease stage V or end stage renal disease |
| 428 | Congestive heart failure, unspecified |
| 428.1 | Left heart failure |
| 428.2 | Systolic heart failure, unspecified |
| 428.21 | Acute systolic heart failure |
| 428.22 | Chronic systolic heart failure |
| 428.23 | Acute on chronic systolic heart failure |
| 428.3 | Diastolic heart failure, unspecified |
| 428.31 | Acute diastolic heart failure |
| 428.32 | Chronic diastolic heart failure |
| 428.33 | Acute on chronic diastolic heart failure |
| 428.4 | Combined systolic and diastolic heart failure, unspecified |
| 428.41 | Acute combined systolic and diastolic heart failure |
| 428.42 | Chronic combined systolic and diastolic heart failure |
| 428.43 | Acute on chronic combined systolic and diastolic heart failure |
| 428.9 | Heart failure, unspecified |
| 492 | Emphysematous bleb |
| 492.8 | Other emphysema |

### Obstructive Sleep Apnea (OSA)

| ICD-9 code | Description |
| --- | --- |
| 327.23 | Obstructive sleep apnea (adult)(pediatric) |
