# Supplementary material for: Risk Factors Associated With Nonfatal Opioid Overdose Leading to Intensive Care Unit Admission: A Cross-sectional Study
Source: JMIR Med Inform. 2021 Nov 8;9(11):e32851. doi: 10.2196/32851 (PMC8663596; doi:10.2196/32851)
Supplement: Multimedia Appendix 2 [file medinform_v9i11e32851_app2.docx]

## Multimedia Appendix 2: NLP Model training and Evaluation

### Task and Experimental Setup

We developed an NLP system to detect SBDH information as well as their *presence* and *change* on word level given an EHR note as input. *Presence* of an SBDH variable reflects its current status with respect to the patient and *Change* is useful to capture any change in the corresponding SBDH variable over time. For example, for the text sequence “Past smoker, stopped”, the word “smoker” represents SBDH variable ‘Smoking’, this activity has no *presence,* and it indicates a *change.* All three tasks were treated as sequence labelling tasks and because they are closely related, we performed multi-task learning to jointly train our NLP model to recognize the SBDH variables, their *presence* (yes, no) and *change* (yes, no) attributes from EHR notes. We considered six types of SBDH variables for this task – 1) Housing insecurity, 2) Unemployment, 3) Social isolation, 4) Alcohol use, 5) Smoking, and 6) Illicit drug use.

### Annotation Process

Our data was annotated by one graduate student and one undergraduate student under the supervision of a senior physician. An inter-annotator agreement (Cohen’s kappa) of 0.898, 0.816 and 0.775 were observed for SBDH, *presence* and *change* attributes, respectively.

We chose the SBDH variables based on the list of social determinants provided by the Kaiser Family Foundation (KFF) [1]. Any indication of lack of housing was annotated as ‘Housing insecurity’ (category ‘Neighborhood and Physical Environment’). Patient's employment status (working currently, lost a job, looking for a job) was considered for annotating ‘Unemployment’ (category ‘Economic Stability’). Lack of social support or community engagement was annotated as ‘Social isolation’ (category ‘Community and Social Context’). Any annotation referring to an event in past was assumed to have attributes - *presence* ‘no’ and *change* ‘yes’ (as shown in the previous example with ‘Smoking’). We did not find any discussion of the SBDH variable ‘Food’ and ‘Healthcare’ in the extracted sections and ‘Education’ had a very low prevalence. Hence, we dropped these variables. In addition, we annotated the mentions and usage of substances - (1) alcohol, (2) tobacco, and (3) illicit drugs to capture behavioral determinants of health.

### NLP Systems and Results

We evaluated several NLP systems using macro average F-score. F-score is the harmonic mean of precision and recall, and a macro metric averages the model performance (F-score) across all the individual classes (e.g., SBDH variables). We did extensive hyperparameter tuning and 10-fold cross-validation for all systems. The train-test split was 80:20. In our experiments, the best system was Bio+Clinical BERT [2], which achieved a macro F-score of 0.86 for SBDH detection with 0.84 macro precision and 0.90 macro recall. For *presence* detection, the model obtained 0.85 macro F-score with 0.83 macro precision and 0.88 macro recall. Finally, for *change* detection, we got macro precision, recall and F1-scores of 0.78, 0.84 and 0.81, respectively.

### Creating SBDH variables

For the first three SBDH variables, we considered the variable value ‘Yes’ if *presence* is ‘yes’, ‘No’ if *presence’* is ‘no’. For the last three SBDH variables, we considered *presence* ‘no’ and *change* ‘no’ as ‘None’, *presence* ‘no’ and *change ‘*yes*’* as ‘Former’, and *presence* ‘yes’ with any prediction for *change* as ‘Current’. In both cases, when the model did not find a particular SBDH variable, we denoted its value as ‘Unknown’. For example, in the previous example with SBDH variable ‘Smoking’, we have ‘no’ *presence* and an indication of *change* (‘yes’), so the variable value will be ‘Former’. It is possible for an EHR note to have mentions of both ‘Former’ and ‘Current’ history of ‘Smoking’ (or ‘Alcohol use’ or ‘Illicit drug use’). In such scenarios, we chose ‘Current’ as the variable value. A brief descriptions and examples of the final set of SBDH variables, used in the nonfatal OD study, have been already provided in Table 1.

## References

1. Heiman HJ, Artiga S. Beyond Health Care : The Role of Social Determinants in Promoting Health and Health Equity What are Social Determinants of Health ? Focus on Health in Non-Health Sectors. Henry J Kaiser Fam Found [Internet] 2015 [cited 2021 Mar 4]; Available from: http://www.ccapcomcare.org/Newsletters/2018-05 INSIGHT KFF Brief.pdf

2. Alsentzer E, Murphy J, Boag W, Weng W-H, Jindi D, Naumann T, McDermott M. Publicly Available Clinical BERT Embeddings. Association for Computational Linguistics (ACL); 2019 [cited 2021 May 31]. p. 72–78. [doi: 10.18653/v1/w19-1909]
